# Sodium Alginate Microencapsulation of an Umami Peptide Fraction (F2) from Goose Bone Paste: Preparation and Reduced Apparent Gastric-Phase Release

**DOI:** 10.3390/foods15152763

**Published:** 2026-08-06

**Authors:** Binghan Chen, Yaguang Xu, Xiuwen Zhang, Feng Lü, Daoying Wang, Ningning Xie, Jingjun Li, Zongyuan Zhen

**Affiliations:** 1 College of Food Science and Engineering, Anhui Science and Technology University, Chuzhou 239000, China; yjs2025412@ahstu.edu.cn (B.C.); yjs2025470@ahstu.edu.cn (X.Z.); yjs2025435@ahstu.edu.cn (F.L.);; 2 Institute of Agro-Product Processing, Anhui Academy of Agricultural Sciences, Hefei 230031, China; 3 Institute of Agricultural Products Processing, Jiangsu Academy of Agricultural Sciences, Nanjing 210014, China; 4 Anhui Provincial Key Laboratory of Functional Agriculture and Functional Foods, Anhui Science and Technology University, Chuzhou 239000, China; 5 Associated Discipline Key Laboratory of Whole Grain Nutrition and High-Value Utilization, Anhui Science and Technology University, Chuzhou 239000, China; 6 The Institute of Functional Agriculture (Food) Science and Technology at Yangtze River Delta (iFAST), Anhui Science and Technology University, Chuzhou 239000, China

**Keywords:** goose bone paste, umami peptide fraction, sodium alginate microencapsulation, ionic gelation, pepsin-free simulated gastric fluid

## Abstract

Goose bone paste is an underutilised poultry-processing by-product and a potential source of taste-active peptides. A nominal 1–3 kDa peptide fraction (F2), operationally designated an umami peptide fraction by analogy with comparable bone-hydrolysate fractions reported in the literature, was isolated from a neutral-protease hydrolysate by sequential ultrafiltration and encapsulated in sodium alginate (SA) microcapsules using extrusion–dripping ionic gelation. Single-factor screening identified the following formulation conditions: 2.0% (*w*/*v*) SA, 2.5% (*w*/*v*) CaCl_2_, 0.3% (*w*/*v*) SE-15, a core-to-wall mass ratio of 0.3, and a preparation temperature of 50 °C. A verification batch prepared under these conditions gave an encapsulation efficiency of 75.44%, with the ±1.07% denoting the SD of three technical determinations from that batch. The dried microcapsules had a moisture content of 2.98 ± 0.21% and passable flowability. The mean particle diameter was 856 ± 52 μm, with a within-batch coefficient of variation of 5.56 ± 0.28%; a complete particle-size distribution was not recorded. In pepsin-free simplified simulated gastric fluid, the apparent release from the microcapsules rose from about 6% at 1 h to about 13% at 5 h, whereas the apparent detection ratio of free F2 rose from about 56% to about 99%. The calculated concentrations fell at or below the validated limit of quantification, the microcapsule-group absorbances lay near the photometric floor of the instrument, and only three sampling times were used. These percentages and the kinetic fits are therefore qualitative to semi-quantitative. The data support only the relative statement that alginate encapsulation lowered the apparent release of F2 under the tested acidic conditions. They do not establish an exact release rate, an error estimate for the microcapsule group, or a specific release mechanism.

## 1. Introduction

The poultry-processing industry generates substantial quantities of by-products, among which bones, cartilage, and connective tissue remain underutilised. Goose bones contain collagen and other proteins that can be enzymatically hydrolysed to produce taste-active peptides with potential applications as natural umami-enhancing food ingredients [1]. Valorising these by-products may simultaneously reduce waste and add value to the food supply chain.

Umami peptides are generally short oligopeptides whose taste activity is influenced by their amino acid composition and sequence, particularly the presence of acidic residues such as Asp and Glu [2,3]. However, food-derived peptides may undergo acid- and enzyme-mediated changes during gastrointestinal transit, potentially affecting their structural integrity and functionality [4]. Delivery systems that limit peptide exposure to adverse gastric conditions are therefore of technological interest.

Microencapsulation is widely used to protect food components from adverse environmental conditions [5,6]. Among the available wall materials, sodium alginate (SA) is attractive because it is food-grade, inexpensive, biocompatible, and capable of forming gels with divalent cations under mild conditions [7]. In the egg-box model, guluronate-rich regions of adjacent alginate chains coordinate Ca^2+^ to form a hydrated three-dimensional network [8]. During extrusion–dripping ionic gelation, an alginate dispersion containing the core material is formed into droplets and introduced into a CaCl_2_ bath. This mild, aqueous process avoids organic solvents and is therefore suitable for thermally and chemically labile peptides. Compared with spray drying, which exposes the core material to heated air, and complex coacervation, which commonly requires careful pH control and may involve an additional cross-linking step, ionic gelation can be conducted under relatively mild processing conditions [8,9]. The morphology, particle size, encapsulation efficiency, and release characteristics of alginate particles can be influenced by the concentrations of alginate and CaCl_2_, the addition of surfactants, and the preparation temperature [9]. For chemically heterogeneous peptide fractions, these properties must be evaluated empirically for each formulation [10].

In the present study, a nominal 1–3 kDa peptide fraction (F2) was isolated from a neutral-protease hydrolysate of goose bone paste and operationally designated an umami peptide fraction by analogy with comparable bone-hydrolysate fractions reported in the literature. F2 was encapsulated in sodium alginate microcapsules using extrusion–dripping ionic gelation. Formulation parameters were screened through single-factor experiments, after which the physicochemical properties and surface morphology of the resulting microcapsules were characterised. Their apparent release behaviour was subsequently evaluated in pepsin-free simplified simulated gastric fluid. This study aimed to obtain formulation, characterisation, and acid-phase release data to inform the development of peptide-loaded food ingredients from poultry-processing by-products.

## 2. Materials and Methods

### 2.1. Materials

Bones were collected from whole carcasses of approximately 10-week-old Landes geese (Anser anser domesticus) obtained from Lu’an Longxiang Meishiwang Poultry Industry Co., Ltd. (Lu’an, Anhui, China). After the adherent muscle and ligaments had been mechanically removed, the bones were rinsed with cold water, drained, and processed using a colloid mill at a bone-to-water ratio of 1:3 (*w*/*v*) until the particle size was ≤2 mm. The resulting goose bone paste was used for subsequent enzymatic hydrolysis without thermal pretreatment.

Sodium alginate (SA; food grade; guluronic acid-to-mannuronic acid ratio, G/M ≈ 1.5; lot no. 20230815) was supplied by Shandong Jiejing Group Co., Ltd. (Qingdao, China). Anhydrous calcium chloride (CaCl_2_; analytical grade) was purchased from Jiangsu Kelongduo Food Ingredients Co., Ltd. (Suqian, China). Sucrose monostearate (SE-15; hydrophilic–lipophilic balance, HLB ≈ 15; food grade) was obtained from Shanghai Chiwei Industrial Co., Ltd. (Shanghai, China). Neutral protease (≥3000 U g^−1^) was supplied by Tianjin Huasheng Chemical Reagent Co., Ltd. (Tianjin, China). All other reagents were of analytical grade.

### 2.2. Enzymatic Hydrolysis of Goose Bone Paste

The goose bone paste suspension was adjusted to pH 7.0 using 1 mol L^−1^ NaOH. Neutral protease was added at 1.5% (*w*/*w*) relative to the mass of the wet goose bone paste. The mixture was hydrolysed at 50 °C for 4 h under continuous magnetic stirring. The enzyme was then inactivated by heating the hydrolysate at 95 °C for 10 min. After cooling to room temperature, the slurry was centrifuged at 8000× *g* for 15 min at 4 °C, and the supernatant was collected as the enzymatic hydrolysate of goose bone paste.

The principal equipment used in this process included an H4-20KR refrigerated high-speed centrifuge (Hunan Kecheng Instrument Equipment Co., Ltd., Changsha, Hunan, China), an SZCL-2 digital temperature-controlled magnetic stirrer with heating (Zhengzhou Kechuang Instrument Co., Ltd., Zhengzhou, Henan, China), and an LGJ-10 vacuum freeze-dryer (Linhai Tanshi Vacuum Equipment Co., Ltd., Linhai, Zhejiang, China). The available model and manufacturer information for the principal equipment is provided in Supplementary Table S2.

### 2.3. Ultrafiltration and Isolation of F2

The hydrolysate was sequentially fractionated using a tangential-flow ultrafiltration system fitted with polyethersulfone membranes with molecular-weight cut-offs (MWCOs) of 3 and 1 kDa. Ultrafiltration was conducted at a transmembrane pressure of 0.2 MPa and a temperature of 25 °C. The material that permeated the 3 kDa membrane but was retained by the 1 kDa membrane was collected as the nominal 1–3 kDa fraction. This fraction was freeze-dried, stored at −20 °C until use, and operationally designated F2.

### 2.4. Preparation of F2 Microcapsules by Extrusion–Dripping Ionic Gelation

F2 microcapsules were prepared by extrusion–dripping ionic gelation. SA was dissolved in deionised water at 2.0% (*w*/*v*), and SE-15 was added at 0.3% (*w*/*v*). F2 was then added at a core-to-wall mass ratio of 0.3, defined as the mass of F2 divided by the mass of SA. Accordingly, the final 100 mL formulation contained 2.0 g of SA, 0.3 g of SE-15, and 0.6 g of F2. The mixture was stirred magnetically at 300 rpm and 50 °C for 30 min.

The resulting 100 mL dispersion was extruded through a 21 G stainless-steel needle with an internal diameter of 0.51 mm at approximately one droplet per second. The vertical distance between the needle tip and the surface of the cross-linking bath was 10 cm. The droplets were introduced into 500 mL of a 2.5% (*w*/*v*) CaCl_2_ solution. Based on the total Ca^2+^ content of the bath and the nominal number of alginate carboxylate groups in the formulation, the bulk molar ratio of Ca^2+^ to –COO^−^ was approximately 11:1. This large nominal excess of Ca^2+^ favoured extensive external ionic cross-linking of the alginate matrix but was not used as direct evidence of complete cross-linking.

After extrusion, the beads were cured in the CaCl_2_ bath for 30 min under gentle stirring at 100 rpm. They were then collected by filtration, rinsed three times with deionised water, and dried at 40 °C for 12 h.

### 2.5. Single-Factor Selection of Formulation Parameters

The effects of five formulation parameters—the core-to-wall mass ratio, SA concentration, preparation temperature, CaCl_2_ concentration, and SE-15 concentration—on the encapsulation efficiency (EE) of the F2 microcapsules were evaluated through sequential single-factor experiments. Seven levels of each factor were tested, as shown in Table 1. Each level was evaluated using three independently prepared batches produced on different days. All batches were prepared using the same lots of SA, F2, and the other reagents. For each independent batch, EE was measured in triplicate, and the mean of the three technical measurements was treated as the result for that batch.

The factors were evaluated sequentially in the following order: core-to-wall mass ratio, SA concentration, preparation temperature, CaCl_2_ concentration, and SE-15 concentration. The selected level of each factor was carried forward to the subsequent experiment. During the initial core-to-wall-ratio experiment, the other factors were fixed at 1.5% (*w*/*v*) SA, 50 °C, 2.0% (*w*/*v*) CaCl_2_, and 0.3% (*w*/*v*) SE-15. During the SA-concentration experiment, the core-to-wall mass ratio was fixed at 0.3, while the preparation temperature, CaCl_2_ concentration, and SE-15 concentration remained fixed at 50 °C, 2.0% (*w*/*v*), and 0.3% (*w*/*v*), respectively. During the preparation-temperature experiment, the core-to-wall mass ratio and SA concentration were fixed at 0.3 and 2.0% (*w*/*v*), respectively, while the CaCl_2_ and SE-15 concentrations were fixed at 2.0% and 0.3% (*w*/*v*), respectively. During the CaCl_2_-concentration experiment, the core-to-wall mass ratio, SA concentration, preparation temperature, and SE-15 concentration were fixed at 0.3, 2.0% (*w*/*v*), 50 °C, and 0.3% (*w*/*v*), respectively. Finally, the SE-15 concentration was evaluated using the selected levels of the other four factors.

This one-factor-at-a-time design was used to select individual factor levels but did not evaluate interactions among the factors. A multivariable optimisation design was not conducted. Each single-factor series was prepared as a separate batch series on different days, so a nominally shared condition appears in more than one series with independently prepared batches. For this reason, EE values are comparable within a series but not across series. A repeated condition, such as 2.0% (*w*/*v*) SA at 50 °C with 0.3 core-to-wall ratio, may therefore give different EE values in different series, and these values are not treated as replicates of a single formulation.

### 2.6. Encapsulation Efficiency

Encapsulation efficiency was calculated using Equation (1):

*EE* (%) = (*m*_1_/*m*_0_) × 100 (1)

where *m*_0_ is the total mass of F2 initially added to the SA–SE-15 formulation and *m*_1_ is the estimated total mass of F2 retained in the recovered fresh microcapsules.

For each determination, 100 mg of freshly prepared wet microcapsules was accurately weighed and mixed with 10.0 mL of 0.1 mol L^−1^ sodium citrate at pH 7.0. The mixture was stirred at 37 °C for 30 min to chelate Ca^2+^ and disrupt the alginate network. The resulting dispersion was centrifuged at 8000× *g* for 10 min, and the absorbance of the supernatant was measured at 280 nm without further filtration or dilution.

The total mass of F2 retained in the recovered microcapsules was estimated from the analysed subsample as follows:

*m*_1_ = *C* × *V* × *D* × (*M_total_*/*M_sample_*)

where *C* is the F2 concentration in the supernatant, *V* is the extraction volume, *D* is the dilution factor (*D* = 1 in this study), *M_total_* is the total fresh mass of microcapsules recovered from the batch, and *M_sample_* is the fresh microcapsule mass used for the determination.

The F2 concentration was calculated using the SGF-based UV calibration curve described in Section 2.10. A separate matrix-matched calibration curve was not prepared for the sodium citrate–alginate–SE-15 matrix, and potential matrix interference at 280 nm was not independently evaluated. The resulting EE values should therefore be interpreted with this analytical limitation in mind.

Three technical determinations were performed for each independent batch, and their mean was used as the EE value for that batch. Three independent batches were prepared for each formulation condition.

### 2.7. Moisture Content

The moisture content of the dried F2 microcapsules was determined gravimetrically. Approximately 2 g of microcapsules that had previously been dried at 40 °C for 12 h were further dried in an oven at 105 °C until a constant mass was reached. Moisture content was calculated using Equation (2):

*Moisture content* (%) = [(*m_a_* − *m_b_*)/*m_a_*] × 100 (2)

where *m_a_* is the mass of the microcapsules before oven drying at 105 °C and *m_b_* is their mass after drying to a constant value. One determination was performed for each of three independently prepared batches, and the results are reported as the mean ± standard deviation (SD) of the three batch-level values.

### 2.8. Angle of Repose, Particle Size, and Coefficient of Variation

The angle of repose (*θ*) of the dried microcapsules was determined using the fixed-funnel method. A separate powder cone was prepared and measured for each of the three independent batches. The angle of repose was calculated using Equation (3):

*tan θ* = *h*/*r* (3)

where *h* is the height of the conical pile and *r* is the radius of its base.

For each independent batch, the diameters of 100 randomly selected microcapsules were measured. The mean particle diameter and SD were calculated separately for each batch. The coefficient of variation (*CV*) of the particle diameter within each batch was calculated using Equation (4):

(4) $$CV\;(\%)=(SD/\bar{d})\;\times \;100$$

where $\bar{d}$ is the mean particle diameter and *SD* is the standard deviation of the 100 diameter measurements within that batch. The overall mean particle diameter was calculated from the three batch-level means. The reported *CV* represents the mean ± SD of the three batch-specific *CV* values.

### 2.9. Surface Morphology

The surface morphology of the dried F2 microcapsules was examined by scanning electron microscopy (SEM; JSM-6380LV, JEOL Ltd., Tokyo, Japan). The samples were mounted on aluminium stubs using double-sided conductive tape and sputter-coated with gold for 60 s. Micrographs were acquired at an accelerating voltage of 20 kV and magnifications of ×43 for the whole-particle overview and ×317 for the surface detail. Imaging was performed at the instrumentation centre of Anhui Science and Technology University.

### 2.10. Apparent Release in Pepsin-Free Simplified Simulated Gastric Fluid

Apparent release was evaluated in a pepsin-free simplified simulated gastric fluid (SGF). The medium was prepared by dissolving NaCl in deionised water at 0.20% (*w*/*v*) and adjusting the pH to 1.2 with concentrated HCl. The medium was maintained at 37 ± 0.5 °C during the experiment. Pepsin was omitted to isolate the response of the calcium alginate matrix to acidic conditions from the effects of enzymatic proteolysis. Consequently, this medium was used as a simplified acid-phase model and was not intended to reproduce complete gastric digestion.

The final 100 mL formulation contained 600 mg of F2. Based on an EE of 75.44%, the estimated mass of F2 retained in the recovered microcapsules was 452.64 mg. After ionic gelation in the CaCl_2_ bath, the beads were collected, washed, and drained of surface liquid. Before any drying, the whole batch of still-wet microcapsules was transferred to a pre-weighed vessel and weighed once. The net fresh mass was 6.00 g. The subsample used for encapsulation efficiency in Section 2.6 and the 200 mg portions placed in the release vessels were both taken from the same freshly prepared, undried microcapsules, so all masses share one fresh-microcapsule basis. This wet mass includes matrix water, bath-derived Ca^2+^ and its bound water, and residual soluble material not fully removed by washing, so the apparent solids content exceeds the 2.9 g of formulation solids fed into the 100 mL dispersion. The value of 75.44 mg g^−1^ is an operational F2 content on a fresh-microcapsule basis rather than a dry-basis content, and the 200 mg portion in each release vessel corresponds to 15.09 mg of F2 on this basis. Because EE and every reported release percentage scale directly with this single fresh-mass determination, the derived masses are treated as operational estimates.

For each of three independent batches prepared on different days, 200 mg of freshly prepared wet F2-loaded microcapsules was dispersed in a separate vessel containing 200 mL of the pepsin-free simplified SGF. A separate vessel containing 15.09 mg of free F2 in 200 mL of the same medium was tested alongside each microcapsule batch. All vessels were stirred magnetically at 60 rpm. Samples were withdrawn sequentially from each vessel at 1, 3, and 5 h. At each time point, a 5 mL aliquot was removed and replaced with an equal volume of prewarmed fresh medium. The aliquots were centrifuged at 8000× *g* for 10 min, and the absorbance of each supernatant was measured once at 280 nm using a UV-1800 UV–visible spectrophotometer (Shimadzu Corporation, Kyoto, Japan).

F2-free simplified SGF was used as the instrumental blank for both the free-F2 and microcapsule groups. Release medium from blank calcium alginate microcapsules was not used for matrix correction. Consequently, potential absorbance from SA, SE-15, or other material released from the microcapsules was not independently subtracted.

#### Calibration and Analytical Limitations

A calibration curve was prepared using freeze-dried F2 dissolved in the simplified SGF at concentrations ranging from 0.12 to 1.20 mg mL^−1^. Each concentration was measured in three technical replicates. The absorbance values were blank-corrected, and the mean absorbance at each concentration was used for regression analysis. The resulting equation was:

*y* = 0.039*x* + 0.0308 (*R*^2^ = 0.9986)

where *x* is the F2 concentration in mg mL^−1^ and *y* is the absorbance at 280 nm.

The limit of quantification (LOQ) was estimated as 10σ/S, where σ was the standard deviation of the regression intercept (0.00039057) and S was the slope of the calibration curve (0.03900673). The calculated LOQ was 0.1001289 mg mL^−1^ and was reported as 0.10 mg mL^−1^.

At the nominal loading used in the release experiment, complete transfer of 15.09 mg of F2 into 200 mL of medium would correspond to approximately 0.075 mg mL^−1^, which is below the estimated LOQ. The concentrations calculated for both the free-F2 and microcapsule groups were therefore obtained by extrapolating the calibration equation below its validated concentration range. Accordingly, the resulting values are reported as semi-quantitative apparent estimates rather than exact F2 concentrations.

UV absorbance at 280 nm primarily reflects aromatic residues such as Tyr, Trp, and Phe and is not specific to intact peptide bonds [11,12]. The term “apparent release” is therefore used for the microcapsule group to describe the proportion of UV280-responsive F2-related material detected in the medium relative to the estimated mass of F2 loaded. For the free-F2 group, “apparent detection ratio” describes the corresponding UV280-based proportion relative to the initially added free F2. These terms do not demonstrate peptide sequence integrity, acid hydrolysis, or retention of sensory activity.

The absorbances underlying the release percentages are small. For the microcapsule group, the blank-corrected readings correspond to roughly 2 × 10^−4^ to 4 × 10^−4^ A, and for the free-F2 group to roughly 2 × 10^−3^ to 3 × 10^−3^ A. The manufacturer states a noise below 5 × 10^−5^ A at 700 nm, a drift below 3 × 10^−4^ A per hour after warm-up, and a photometric accuracy within ±0.002 A for the UV-1800. The microcapsule-group signal and its batch-to-batch variation therefore lie at or below the instrument noise and drift, and the free-F2 signal lies near the photometric-accuracy limit. Each supernatant was measured once, and no raw absorbance dataset with independent replication at each time point is available for these readings. The microcapsule-group release values are consequently reported without error bars or a standard deviation and are treated as qualitative indices. The free-F2 values are treated as semi-quantitative. No significance test is applied to the microcapsule group.

Apparent release at each sampling time was calculated with a correction for the analyte removed in preceding aliquots, using Equation (5):

Apparent release (%) = [(C_n_V + Σ_i=1_^n−1^ C_i_V_s_)/m_F2_] × 100 (5)

where C_n_ is the calculated F2 concentration at the nth sampling time, V is the total volume of release medium (200 mL), V_s_ is the volume removed and replaced at each preceding sampling time (5 mL), C_i_ represents the calculated concentrations at the preceding sampling times, and m_F2_ is the estimated mass of F2 loaded into the vessel. The aliquot volume represented 2.5% of the total medium volume.

For the microcapsule group, m_F2_ was estimated from the initial F2 mass, the measured EE, and the total recovered wet microcapsule mass. For the free-F2 group, m_F2_ was the mass of free F2 added directly to each vessel. Because the calculated concentrations were below the estimated LOQ and blank microcapsules were not used for matrix correction, the percentages derived from Equation (5) should be interpreted as semi-quantitative apparent indices.

### 2.11. Exploratory Fitting of Release Models

The mean cumulative apparent release values for the microcapsule group at 1, 3, and 5 h were fitted to four release models, including the Korsmeyer–Peppas model [13], using Origin 2024 (OriginLab Corporation, Northampton, MA, USA). Nonlinear least-squares fitting was performed on the original apparent-release scale. The models were defined as follows:

*Q_t_* = *k*_0_*t* (6)

*Q_t_* = 100[1 − *exp*(−*k*_1_*t*)] (7)

*Q_t_* = *k_H_t*^1/2^ (8)

*Q_t_*/100 = *k_KP_t^n^* (9)

where *Q_t_* is the cumulative apparent release (%) at time *t*; *k*_0_, *k*_1_, *k_H_*, and *k_KP_* are the fitted rate constants for the zero-order, first-order, Higuchi, and Korsmeyer–Peppas models, respectively; and *n* is the Korsmeyer–Peppas exponent. The zero-order and Higuchi models were constrained to pass through the origin. For the first-order model, complete release was fixed at 100%. The Korsmeyer–Peppas model was fitted on the original proportional scale rather than after logarithmic transformation. *R*^2^ was calculated on the original apparent-release scale.

Only the three mean values at 1, 3, and 5 h were used for fitting. Because the fits were based on three time points and on semi-quantitative estimates below the LOQ, the fitted parameters were used only for exploratory description. The models were not ranked, and neither *R*^2^ nor the Korsmeyer–Peppas exponent was used to infer a release mechanism.

### 2.12. Statistical Analysis

Unless otherwise specified, data are reported as the mean ± SD of three independent batches prepared on different days using the same lots of SA, F2, and the other reagents. Technical replicate measurements were averaged within each batch before statistical analysis, and the independent batch was treated as the experimental unit.

For the single-factor experiments, differences among the seven levels of each factor were evaluated by one-way analysis of variance followed by Duncan’s multiple range test using SPSS Statistics version 25.0 (IBM Corp., Armonk, NY, USA). Differences were considered statistically significant at *p* < 0.05. Different lowercase letters indicate significant differences among the levels of the corresponding factor.

For the release experiment, each supernatant was measured once, and the microcapsule-group absorbances lay at or below the instrument noise and drift. No error estimate or significance test is reported for the microcapsule group, and its values are presented qualitatively. The free-F2 values, whose absorbances lay near the photometric-accuracy limit, are reported as semi-quantitative means of three independent release experiments without inferential testing. Formal group comparison was not performed, because the underlying microcapsule-group readings are not resolvable on the instrument used.

**Figure 1:**
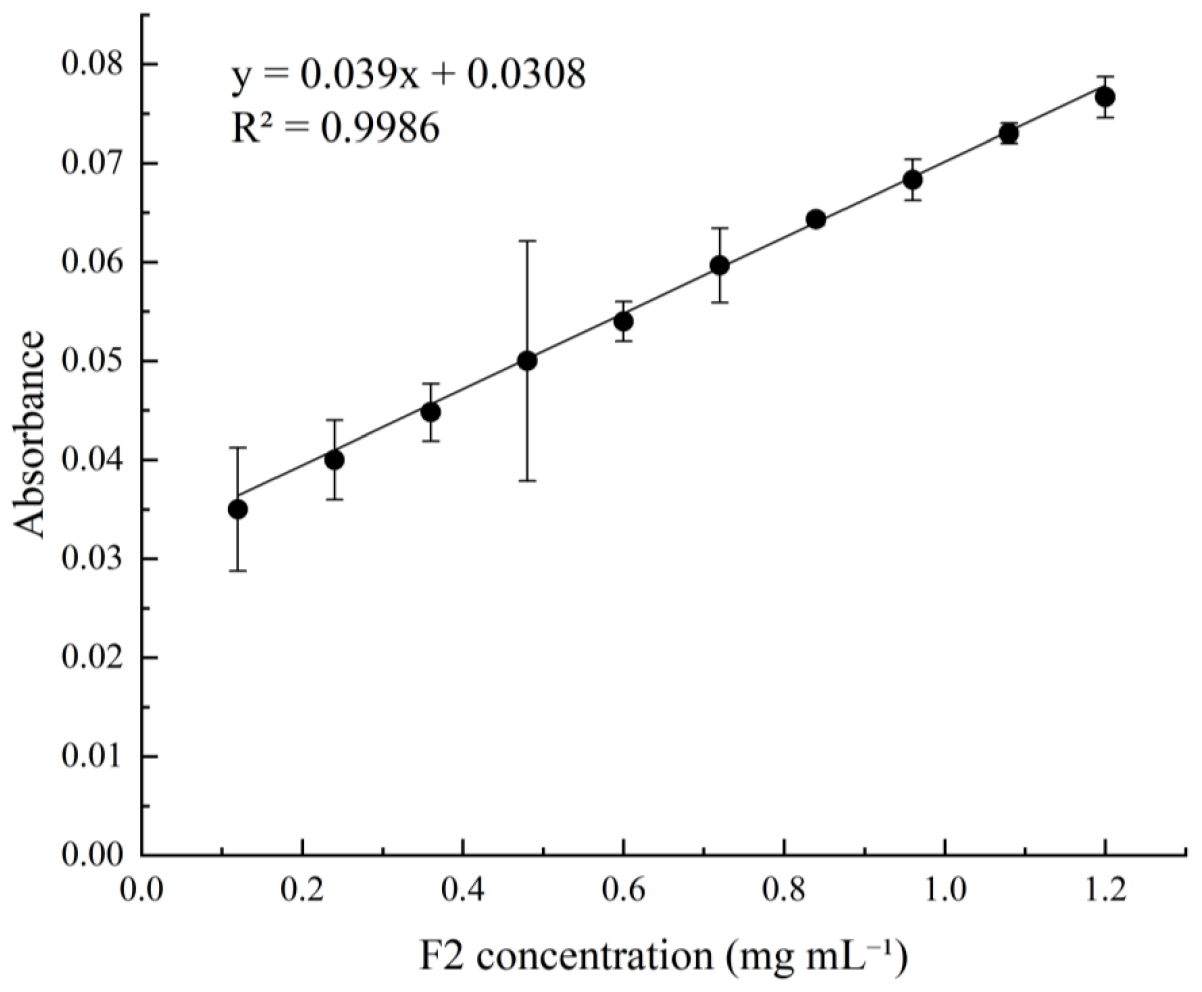
Calibration curve for F2 measured by UV absorbance at 280 nm. F2 standards were prepared in pepsin-free simplified simulated gastric fluid over the range of 0.12–1.20 mg mL^−1^. Data are presented as the mean ± SD of three technical measurements at each concentration. The regression was fitted to the mean absorbance values: *y* = 0.039*x* + 0.0308, *R*^2^ = 0.9986. The estimated LOQ was 0.10 mg mL^−1^.

For the calibration curve in Figure 1, each concentration was measured in three technical replicates. These technical measurements were used to estimate the mean and SD at each concentration and were not treated as independent experimental batches. Graphs were prepared using Origin 2024.

## 3. Results and Discussion

### 3.1. Literature Context and Operational Designation of F2

Fraction F2 was operationally defined as the nominal 1–3 kDa ultrafiltration fraction used in the present encapsulation study. It was analysed and encapsulated as a bulk fraction rather than as a single peptide or a chemically defined mixture. Previous studies provide a relevant context for this operational designation: YDAELS, TDVAHR, and ELELQ were identified as umami-active peptides in bovine bone soup, whereas taste-active peptides have also been characterised from fermented goose bone [14,15]. However, the peptide sequences, amino acid composition, and sensory properties of F2 were not determined in the present study. The findings cited above therefore support the broader bone-derived-peptide context but do not establish that F2 contains the same sequences or has experimentally verified umami activity.

### 3.2. Effects of Formulation Parameters on Encapsulation Efficiency

**Figure 2:**
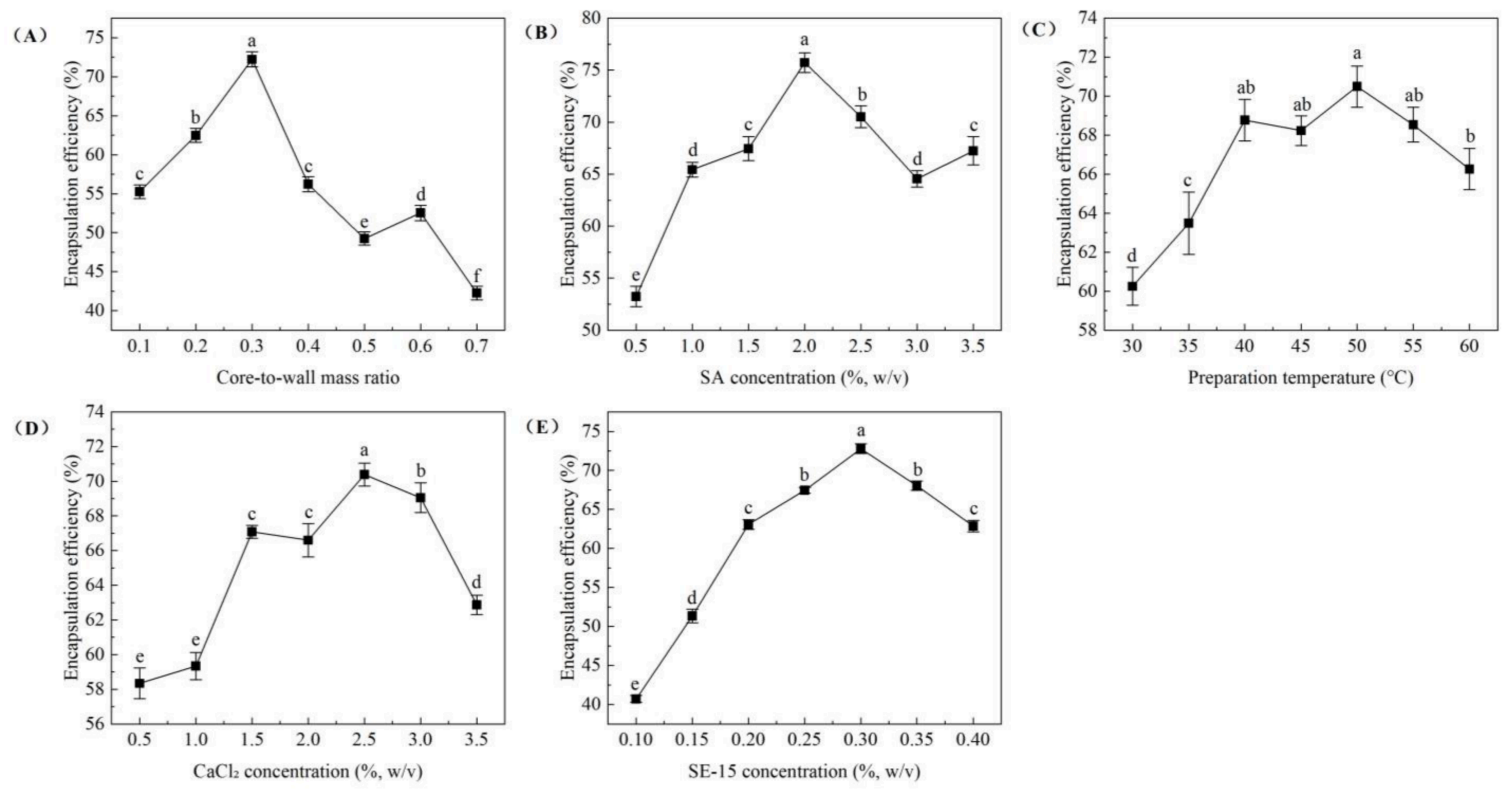
Effects of (**A**) core-to-wall mass ratio, (**B**) sodium alginate (SA) concentration, (**C**) preparation temperature, (**D**) CaCl_2_ concentration, and (**E**) SE-15 concentration on the encapsulation efficiency (EE) of F2 microcapsules during sequential single-factor screening. Data are presented as the mean ± SD of three independently prepared batches. For each batch, three technical determinations were averaged before calculation of the batch-level mean. Different lowercase letters indicate significant differences among the seven levels within the corresponding panel (*p* < 0.05; one-way ANOVA followed by Duncan’s multiple range test). Comparisons of letters are valid within, but not between, panels.

The effects of the five formulation parameters on the encapsulation efficiency (EE) of F2 microcapsules are shown in Figure 2. Each plotted value represents the mean ± SD of three independently prepared batches, with the technical measurements first averaged within each batch.

As the core-to-wall mass ratio increased from 0.1 to 0.3, EE increased from 55.25 ± 0.88% to 72.21 ± 0.96%. Further increases in the ratio reduced EE, and the lowest value, 42.26 ± 0.86%, was observed at a ratio of 0.7 (Figure 2A). This pattern indicates that a core-to-wall mass ratio of 0.3 was the most suitable among the levels tested. Although the decline at higher ratios may reflect insufficient alginate relative to the amount of F2, the present experiment did not directly measure the distribution or loss of unencapsulated F2 during bead formation.

EE also varied with SA concentration, reaching its highest value at 2.0% (*w*/*v*) SA (75.72 ± 0.96%; Figure 2B). Lower concentrations may have provided less polymer for retaining F2, whereas higher concentrations could have altered the viscosity and droplet-forming behaviour of the formulation [16]. However, viscosity and droplet-formation dynamics were not measured; these explanations should therefore be regarded as plausible interpretations rather than demonstrated mechanisms.

Preparation temperature produced a comparatively moderate response, with EE increasing from 60.25 ± 0.98% at 30 °C to 70.49 ± 1.06% at 50 °C before decreasing at 55 and 60 °C (Figure 2C). The highest EE among the tested temperatures was therefore obtained at 50 °C. This result identifies an appropriate processing temperature for the present formulation but does not demonstrate a temperature-dependent molecular mechanism.

For CaCl_2_, EE reached its highest value at 2.5% (*w*/*v*) (70.39 ± 0.66%; Figure 2D). Calcium concentration affects alginate gelation and bead properties, but these effects depend on alginate composition and processing conditions [17]. The lower EE values observed below and above 2.5% (*w*/*v*) therefore indicate a formulation-specific response and should not be interpreted as evidence that 2.5% (*w*/*v*) is universally optimal for alginate encapsulation.

Finally, EE increased from 40.70 ± 0.43% at 0.10% (*w*/*v*) SE-15 to 72.79 ± 0.61% at 0.30% (*w*/*v*), before decreasing to 62.87 ± 0.72% at 0.40% (*w*/*v*) (Figure 2E). Accordingly, 0.30% (*w*/*v*) was selected as the SE-15 concentration for the combined formulation.

A separate verification batch prepared using the selected levels—a core-to-wall mass ratio of 0.3, 2.0% (*w*/*v*) SA, a preparation temperature of 50 °C, 2.5% (*w*/*v*) CaCl_2_, and 0.30% (*w*/*v*) SE-15—had an EE of 75.44 ± 1.07%. This value is the mean ± SD of three technical determinations from one verification batch and should not be interpreted as the mean of three independently prepared verification batches. The verification value was close to the highest values observed during single-factor screening; however, no statistical comparison was performed between the verification batch and the single-factor experiments.

The single-factor design identified suitable levels within the ranges examined but did not evaluate interactions among the five factors or establish a global optimum. Response-surface or factorial experiments would be required to assess interactions and formally optimise the formulation. Direct quantitative comparison with previous peptide-encapsulation studies is also limited by differences in core materials, wall systems, preparation methods, and definitions of EE. Garzón et al. investigated the effects of agar–carrageenan wall composition and core-to-wall ratio on microencapsulated bioactive peptides [18], whereas Tian et al. used a starch sodium octenylsuccinate/SA system to prepare wheat-germ albumin polypeptide microcapsules [19]. These studies provide methodological context but do not establish a universal EE range directly applicable to the present F2–alginate system.

### 3.3. Role of SE-15 in Formulation Performance

SE-15 is a hydrophilic, non-ionic sucrose fatty-acid ester included in the formulation as a surfactant. In the single-factor experiment, EE increased as the SE-15 concentration rose from 0.10% to 0.30% (*w*/*v*) and decreased at concentrations above 0.30% (*w*/*v*). Thus, 0.30% (*w*/*v*) was the most suitable concentration among the levels tested.

The present data demonstrate an association between SE-15 concentration and EE but do not identify the underlying mechanism. Surface tension, formulation viscosity, droplet shape, interfacial behaviour, Ca^2+^ diffusion, matrix porosity, and SE-15 retention were not measured. It would therefore be premature to attribute the EE response specifically to improved droplet sphericity, reduced tailing, altered Ca^2+^ diffusion, delayed interfacial cross-linking, or changes in wall porosity. These possibilities could be evaluated in future studies using direct measurements of surface tension, rheological properties, droplet morphology, swelling, and microcapsule porosity.

### 3.4. Physicochemical Properties of F2 Microcapsules

The physicochemical properties of the dried F2 microcapsules prepared using the selected formulation are presented in Table 2. Their moisture content was 2.98 ± 0.21%. Although this relatively low value indicates that little residual water remained after drying, moisture content alone is insufficient to establish storage or microbial stability. Water activity, which was not measured in the present study, would be required for a more direct assessment of microbial stability [20].

The angle of repose was 43.60 ± 0.36°, corresponding to passable flowability according to commonly used powder-flow classifications [21]. This result describes the flow behaviour under the conditions of the fixed-funnel test but does not establish performance during large-scale conveying, mixing, or filling.

The mean particle diameter was 856 ± 52 μm. The mean within-batch coefficient of variation was 5.56 ± 0.28%, indicating relatively limited diameter variation among the 100 particles measured within each batch. However, a complete particle-size distribution was not recorded; therefore, the CV should not be interpreted as evidence that the formulation would necessarily exhibit uniform release behaviour or equivalent performance in different food matrices.

### 3.5. Surface Morphology and Apparent Release in Simplified SGF

**Figure 3:**
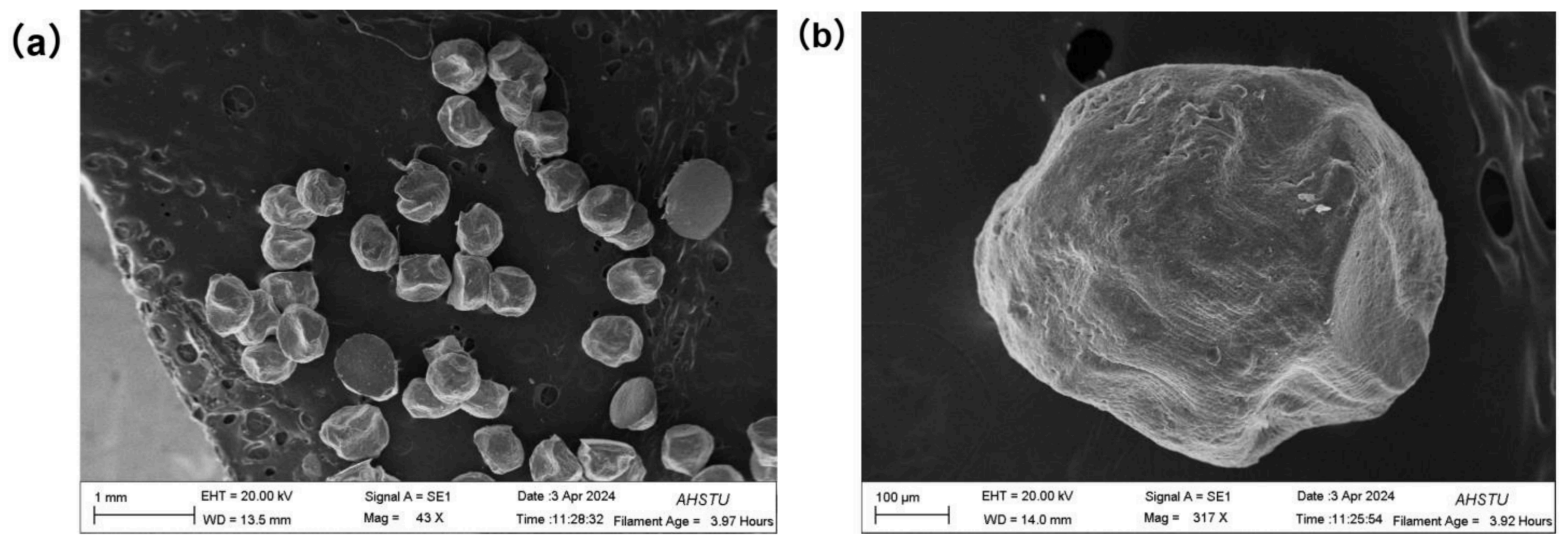
Scanning electron micrographs of dried F2 microcapsules prepared using a core-to-wall mass ratio of 0.3, 2.0% (*w*/*v*) sodium alginate, 2.5% (*w*/*v*) CaCl_2_, 0.30% (*w*/*v*) SE-15, and a preparation temperature of 50 °C: (**a**) overview of multiple particles at ×43 magnification; (**b**) higher-magnification image of a selected particle at ×317. The scale bars represent 1 mm in (**a**) and 100 μm in (**b**), respectively.

The SEM images of the dried F2 microcapsules are shown in Figure 3. At ×43 magnification, the particles appeared generally rounded but exhibited irregular outlines and some deformation (Figure 3a). The ×317 image showed a heterogeneous surface with ridges, depressions, and locally folded regions (Figure 3b). No obvious large fracture was visible in the selected area of the particle shown at higher magnification. However, the images do not establish that every microcapsule possessed a complete, continuous shell, nor do they provide quantitative information on surface porosity, shell thickness, or internal structure.

**Figure 4:**
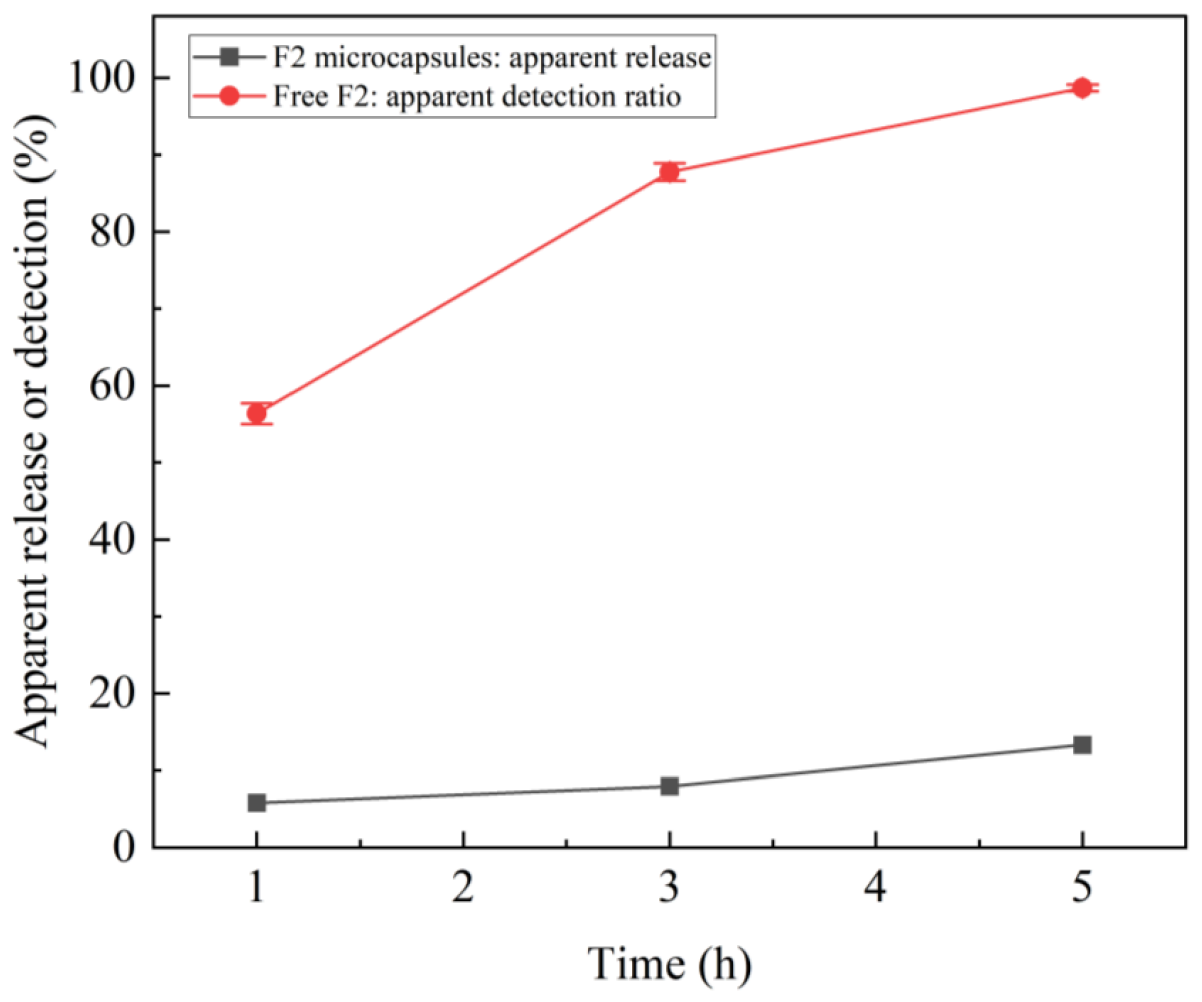
Apparent release from F2-loaded microcapsules and apparent detection ratio of free F2 in pepsin-free simplified simulated gastric fluid at pH 1.2 and 37 °C. Free-F2 values are semi-quantitative means of three independent release experiments. Microcapsule values are shown without error bars, because the corresponding absorbances lay at or below the instrument noise and drift. No significance test is applied. The calculated concentrations were at or below the validated LOQ, so the percentages indicate a direction of difference rather than resolved quantities.

Figure 4 compares the apparent release from the F2-loaded microcapsules with the apparent detection ratio of free F2 in pepsin-free simplified SGF. The free-F2 apparent detection ratio rose from about 56% at 1 h to about 88% at 3 h and about 99% at 5 h. The microcapsule apparent-release estimates rose from about 6% at 1 h to about 8% at 3 h and about 13% at 5 h. The microcapsule values were lower than the free-F2 values at every sampling time. The microcapsule-group absorbances lay at or below the instrument noise and drift, so these values are shown without error bars and are read only as a direction of difference, not as resolved quantities.

These comparisons indicate that less UV280-responsive F2-related material was detected from the microcapsule vessels than from the free-F2 vessels under the tested acidic conditions. However, complete transfer of the nominal F2 dose into the release medium would have produced a concentration below the calibrated LOQ of 0.10 mg mL^−1^. All release-study estimates were therefore obtained by extrapolating the calibration equation below its validated range. In addition, the UV280 assay was not specific to intact F2 peptides, and release medium from blank alginate microcapsules was not used for matrix correction. Consequently, the reported percentages should be interpreted as semi-quantitative apparent indices rather than exact release or dissolution measurements. The free-F2 apparent detection ratio was below the added mass at 1 h and approached completeness only at 5 h. Complete transfer of 15.09 mg into 200 mL corresponds to about 0.075 mg mL^−1^, which is below the validated LOQ of 0.10 mg mL^−1^, so early readings taken near the analytical floor systematically underestimate the dissolved fraction. Slow or incomplete dissolution and possible transient aggregation of the peptide fraction at pH 1.2 may add to this early shortfall. The two contributions cannot be separated with the present single-wavelength, below-LOQ measurements.

No obvious macroscopic disintegration of the microcapsules was noted during visual observation of the acidic-medium experiment. This observation was not quantified, and particle size, swelling, mass loss, and structural integrity were not measured during incubation. It therefore cannot be used to establish the absence of swelling or to define the mechanism responsible for the lower apparent-release values.

The mean apparent-release estimates at 1, 3, and 5 h were fitted to four release models, and the resulting parameters are presented in Supplementary Table S1. Because only three mean time points were available and the underlying values were below the validated LOQ, the fits are exploratory. The models should not be ranked on the basis of their *R*^2^ values, and the Korsmeyer–Peppas exponent should not be used to assign a release mechanism.

An intestinal phase was not included in the experiment. The present results therefore do not demonstrate that the microcapsules would swell, disintegrate, or release F2 at intestinal pH. Sequential gastric- and intestinal-phase experiments, together with quantitative measurements of particle swelling, mass loss, and peptide integrity, are required to evaluate gastrointestinal release behaviour.

## 4. Conclusions

In this study, a nominal 1–3 kDa fraction, operationally designated F2, was isolated from a neutral-protease hydrolysate of goose bone paste and encapsulated in sodium alginate microcapsules by extrusion–dripping ionic gelation. Sequential single-factor screening identified the following selected levels within the ranges tested: a core-to-wall mass ratio of 0.3, 2.0% (*w*/*v*) SA, a preparation temperature of 50 °C, 2.5% (*w*/*v*) CaCl_2_, and 0.30% (*w*/*v*) SE-15. One verification batch prepared using these levels had an EE of 75.44 ± 1.07%, reported as the mean ± SD of three technical determinations. Because factor interactions were not evaluated, these conditions should not be interpreted as a globally optimised formulation.

The dried microcapsules had a moisture content of 2.98 ± 0.21%, an angle of repose of 43.60 ± 0.36°, a mean particle diameter of 856 ± 52 μm, and a mean within-batch particle-diameter CV of 5.56 ± 0.28%. SEM showed generally rounded particles with irregular outlines and heterogeneous surface features. The images did not establish shell continuity, porosity, or internal structure.

In pepsin-free simplified SGF at pH 1.2 and 37 °C, the apparent-release estimate for the encapsulated group rose from about 6% at 1 h to about 13% at 5 h, whereas the apparent detection ratio of free F2 rose from about 56% to about 99%. The lower values for the microcapsule group support the relative conclusion that alginate encapsulation reduced the amount of UV280-responsive F2-related material detected under the tested acidic conditions. They do not establish an exact release rate, preservation of intact peptide sequences, retention of sensory activity, or a specific release mechanism.

The study has several analytical and experimental limitations. All calculated release-study concentrations were below the validated LOQ, the UV280 assay was not specific to intact F2 peptides, and blank alginate microcapsules were not used for matrix correction. The exploratory kinetic fits were based on only three mean time points. In addition, water activity, formulation viscosity, surface tension, swelling, mass loss, porosity, shell thickness, peptide composition, and sensory activity were not measured. The experiment did not include pepsin or an intestinal phase and therefore did not reproduce complete gastrointestinal digestion.

Future work should use a validated, matrix-matched analytical method with an appropriate quantification range and should evaluate peptide identity and integrity. Factorial or response-surface designs could be used to assess interactions among formulation variables. Quantitative measurements of water activity, rheological properties, particle-size distribution, swelling, mass loss, and storage stability would strengthen physicochemical characterisation. Sequential gastric- and intestinal-phase experiments, including an appropriate INFOGEST-based design, are required before claims can be made regarding gastrointestinal protection or intestinal release.

## Figures and Tables

**Table 1:** Levels of the five formulation variables evaluated during single-factor screening of F2 microcapsules.

Factor	Level 1	Level 2	Level 3	Level 4	Level 5	Level 6	Level 7	Selected Level
Core-to-wall mass ratio	0.1	0.2	0.3	0.4	0.5	0.6	0.7	0.3
SA concentration (%, *w*/*v*)	0.5	1.0	1.5	2.0	2.5	3.0	3.5	2.0
Preparation temperature (°C)	30	35	40	45	50	55	60	50
CaCl_2_ concentration (%, *w*/*v*)	0.5	1.0	1.5	2.0	2.5	3.0	3.5	2.5
SE-15 concentration (%, *w*/*v*)	0.10	0.15	0.20	0.25	0.30	0.35	0.40	0.30

Note: The factors were evaluated sequentially. The selected level of each factor was used in the subsequent experiments. Before evaluation, SA and CaCl_2_ were initially fixed at 1.5% and 2.0% (*w*/*v*), respectively; the preparation temperature and SE-15 concentration were fixed at 50 °C and 0.3% (*w*/*v*), respectively. The selected SA concentration of 2.0% (*w*/*v*) was used in the subsequent temperature, CaCl_2_, and SE-15 experiments, and the selected CaCl_2_ concentration of 2.5% (*w*/*v*) was used in the final SE-15 experiment.

**Table 2:** Physicochemical properties of dried F2 microcapsules prepared using the selected formulation.

Property	Value
Moisture content (%)	2.98 ± 0.21
Angle of repose (°)	43.60 ± 0.36
Mean particle diameter (μm)	856 ± 52
Coefficient of variation of particle diameter (%)	5.56 ± 0.28

Note: Data are presented as the mean ± SD of three independently prepared batches. Moisture content and angle of repose were determined once for each batch. For particle-size analysis, 100 microcapsules were measured per batch. A mean diameter and a within-batch coefficient of variation were calculated for each batch; the table reports the mean ± SD of the three batch-level values.

## Data Availability

The original contributions presented in this study are included in the article/Supplementary Materials. Further inquiries can be directed to the corresponding author.

## Supplementary Materials

The following supporting information can be downloaded at https://www.mdpi.com/article/10.3390/foods15152763/s1: Table S1: Exploratory fitting results of release models for encapsulated F2 in simulated gastric fluid; Table S2: Available information on the principal equipment used in this study.

## Abbreviations

The following abbreviations are used in this manuscript:

ANOVA	analysis of variance
CV	coefficient of variation
EE	encapsulation efficiency
MWCO	molecular-weight cut-off
SA	sodium alginate
SE-15	sucrose monostearate
SEM	scanning electron microscopy
SGF	simulated gastric fluid
