# Supplementary material for: Sodium Alginate Microencapsulation of an Umami Peptide Fraction (F2) from Goose Bone Paste: Preparation and Reduced Apparent Gastric-Phase Release
Source: Foods. 2026 Aug 6;15(15):2763. doi: 10.3390/foods15152763 (PMC13465211; doi:10.3390/foods15152763)
Supplement: Supplementary file 1 [file foods-15-02763-s001.zip › foods-4419874-supplementary.pdf]

Supplementary Materials

Table S1. Exploratory fitting results of release models for encapsulated F2 in simulated gastric fluid

Release model	Model equation	Fitted parameter(s)	R^2
Zero-order	$Q_t = k_0 t$	$k_0 = 2.744\%\ h^{-1}$	0.670
First-order	$Q_t = 100[1 - \exp(-k_1 t)]$	$k_1 = 0.0293\ h^{-1}$	0.697
Higuchi	$Q_t = k_H t^{1/2}$	$k_H = 5.474\%\ h^{-1/2}$	0.879
Korsmeyer–Peppas	$Q_t/100 = k_{KP} t^n$	$k_{KP} = 0.0497\ h^{-n}$; $n = 0.576$	0.890

Note: The models were fitted in Origin 2024 by nonlinear least squares using only the three mean cumulative apparent release values at 1, 3, and 5 h: 5.82%, 7.94%, and 13.28%, respectively. The zero-order and Higuchi models were constrained to pass through the origin. Complete release in the first-order model was fixed at 100%. The Korsmeyer–Peppas model was fitted on the original proportional scale, and R^2 was calculated on the original apparent-release scale. Because only three time points were available and all calculated concentrations were below the estimated LOQ, the parameters are exploratory and were not used to rank the models or assign a release mechanism. Because the microcapsule-group release values are reported qualitatively and without an error estimate, the fitted constants and R^2 are descriptive only and are not used to compare models or to infer a mechanism.

Table S2. Available information on the principal equipment used in this study

Instrument/equipment	Model	Manufacturer	Location
Refrigerated high-speed centrifuge	H4-20KR	Hunan Kecheng Instrument Equipment Co., Ltd.	Changsha, Hunan, China
Digital temperature-controlled magnetic stirrer with heating	SZCL-2	Zhengzhou Kechuang Instrument Co., Ltd.	Zhengzhou, Henan, China
Vacuum freeze-dryer	LGJ-10	Linhai Tanshi Vacuum Equipment Co., Ltd.	Linhai, Zhejiang, China
pH meter	P901	Shanghai Youke Instrument and Meter Co., Ltd.	Shanghai, China
Analytical balance	FA224	Shanghai Lichen-BX Instrument Technology Co., Ltd.	Shanghai, China
UV–Vis spectrophotometer	UV-1800	Shimadzu Corporation	Kyoto, Japan

Instrument/equipment	Model	Manufacturer	Location
Scanning electron microscope	JSM-6380LV	JEOL Ltd.	Tokyo, Japan

Note: Only equipment for which model and/or manufacturer information could be verified from the available records is listed. For several legacy items used in the study, including the colloid mill, tangential-flow ultrafiltration system and associated 1 and 3 kDa membranes, drying oven, and optical micrometer, the model, manufacturer, supplier, or catalogue information could not be recovered because the relevant equipment identification or archived records were incomplete. No unverified model or manufacturer has been assigned.
